# Optimal Timing of Serum Lipase for Early Prediction of Clinically Relevant Pancreatic Fistula After Pancreaticoduodenectomy

**DOI:** 10.3390/cancers18172821

**Published:** 2026-09-01

**Authors:** Roberto Cammarata, Ludovico Carbone, Vincenzo La Vaccara, Roberto Coppola, Damiano Caputo

**Affiliations:** 1 Operative Research Unit of General Surgery, Fondazione Policlinico Universitario Campus Bio-Medico, 00128 Rome, Italy; roberto.cammarata@policlinicocampus.it (R.C.); r.coppola@policlinicocampus.it (R.C.); d.caputo@unicampus.it (D.C.); 2 Research Unit of General Surgery, Università Campus Bio-Medico di Roma, Via Álvaro del Portillo, 21, 00128 Rome, Italy; ludovicocarbone1@gmail.com

**Keywords:** pancreatic cancer, pancreaticoduodenectomy, pancreatic fistula, lipase, procalcitonin, biomarkers

## Abstract

**Background**: Pancreatic fistula remains the leading cause of morbidity after pancreaticoduodenectomy. However, its diagnosis is frequently delayed. We evaluate the predictive accuracy of serum lipase in detecting a clinically relevant pancreatic fistula (CR-POPF) and assess whether its combination with procalcitonin (PCT) improves risk stratification. **Methods**: A single-center study was conducted that included 115 patients who underwent pancreaticoduodenectomy between 2015 and 2019. Serum lipase levels were measured preoperatively and on postoperative day (POD) 1, 2, and 3. Elevated lipase was defined as >3× the institutional upper limit of normal (>177 U/L). **Results**: Serum lipase levels were higher in patients who developed CR-POPF at all postoperative days (*p* < 0.01). The area under the curve (AUC) increased from POD 1 (0.76) to POD 2 (0.82) and remained stable at POD 3 (0.82), while specificity improved from 52.7% to 89.2%, with consistently high negative predictive values (92.5–93.3%). Elevated lipase on POD 3 predicts CR-POPF (OR 6.89, 95% CI 1.61–29.53; *p* = 0.009). The combination of elevated lipase, regardless of the preoperative value, and PCT ≥ 1.5 ng/mL on POD 3 achieved full specificity. **Conclusions**: Early postoperative serum lipase levels are useful to exclude CR-POPF after pancreaticoduodenectomy. POD 3 provided a proper diagnostic value. The combination of serum lipase and PCT on POD 3 may further improve accuracy, enabling tailored postoperative surveillance.

## Simple Summary

Postoperative pancreatic fistula can lead to serious infections, bleeding, and even death. Early identification of patients at risk to develop a pancreatic fistula following pancreaticoduodenectomy remains a major challenge. This study investigates the accuracy of lipase serum levels during the first three days after surgery in predicting which patients are most likely to develop a clinically relevant fistula (grade B/C), to optimize their management. Our results show that serum lipase measured on the third day after surgery is the most accurate predictor, and that its combination with normal procalcitonin levels further improves specificity, potentially identifying patients at very low risk to develop a pancreatic fistula.

## 1. Introduction

Pancreaticoduodenectomy (PD) is the cornerstone treatment for pancreatic head and periampullary malignancies. Despite advances in surgical technique and perioperative care, it is still associated with a major complication rate of approximately 26% and a 90-day mortality of 5%, even in high-volume centers [1,2].

Postoperative morbidity following PD is largely driven by a concomitant postoperative pancreatic fistula (POPF). Clinically relevant POPF (CR-POPF), graded as B or C, occurs in up to 14% of patients and may lead to severe complications, including hemorrhage, intra-abdominal infection, and organ failure [3]. As a consequence, early identification of patients at risk for CR-POPF remains a major priority in pancreatic surgery [4].

Since the International Study Group of Pancreatic Surgeons (ISGPS) standardized the definition and grading of POPF [5], the understanding of its pathophysiology has evolved beyond a simple anastomotic leak. Particularly, increasing evidence suggests that an acute inflammatory process of the pancreatic remnant, initially termed postoperative acute pancreatitis or POAP and later defined by the ISGPS as post-pancreatectomy acute pancreatitis or PPAP [6,7], may precede and contribute to the fistula development. PPAP is defined by sustained postoperative hyperamylasemia with clinical and radiological findings [8]. However, the requirement for all diagnostic criteria may underestimate its true incidence [9]. Moreover, postoperative elevation in pancreatic enzymes may reveal different mechanisms, including surgical trauma, injury after ischemia and reperfusion, and preserved exocrine function [6].

Recent evidence suggests that serum lipase measured on postoperative day (POD) 1 outperforms amylase in predicting CR-POPF [10,11], although its clinical role is still unclear. The rationale behind the development of CR-POPF is thought to involve persistent inflammation and injury of the pancreatic remnant, which results from pancreatic ischemia, surgical manipulation, and impaired healing of the pancreatic anastomosis. In this setting, ongoing acinar cell injury and pancreatic inflammation may lead to persistent release of pancreatic enzymes into the circulatory system. In a previous collaboration with microbiologists, procalcitonin (PCT) has been shown to reflect persistent systemic inflammation and to identify patients at higher risk of clinically relevant fistula [12]. The potential value of PCT may be related to its ability to indicate a systemic component of the pathological process, thereby providing complementary information to pancreatic enzyme levels. The present study aims to determine the optimal timing of perioperative serum lipase measurement for early prediction of CR-POPF. As a secondary aim, we evaluated whether combining serum lipase at its most informative time point with postoperative PCT could further improve patients’ risk to develop a fistula.

## 2. Materials and Methods

### 2.1. Study Design

This was a retrospective, single-center study. Patients undergoing PD between January 2015 and December 2019 in the Department of General Surgery at the Fondazione Policlinico Universitario Campus Bio-Medico were included.

Specifically, cancer patients affected by pancreatic ductal adenocarcinoma, distal cholangiocarcinoma, adenocarcinoma of the ampulla of Vater, or duodenal adenocarcinoma and whose serum lipase levels were measured in the first three days after surgery were enrolled. All consecutive patients meeting the inclusion criteria were considered for enrollment. To better reflect clinical practice, patients receiving neoadjuvant therapy were not excluded. Exclusion criteria were as follows: (1) clinical infection at the time of surgery, (2) autoimmune disorders or hematological malignancies, (3) emergency surgery, (4) age younger than 18 years, and (5) distant metastasis at the time of diagnosis or unresectable cancer at presentation.

### 2.2. Variables

Demographic characteristics (age, sex, BMI, and ASA score), laboratory tests, definitive histopathological diagnosis, intraoperative data (i.e., assessment of the main pancreatic duct size and pancreatic gland texture), and length of hospital stay were collected.

Postoperative complications were recorded, including POPF, biliary fistula, enteric fistula, intra-abdominal collection, intra-abdominal hemorrhage, delayed gastric emptying, sepsis, and surgical site infection, and were classified according to the Clavien–Dindo classification, along with 90-day mortality.

Soft pancreatic texture was defined as friable parenchyma, as assessed intraoperatively by the operating surgeon [13]. Even in the presence of a dilated main pancreatic duct, pancreatic texture was classified as soft when no signs of parenchymal inflammation or fibrosis were identified intraoperatively. CR-POPF was defined according to the 2016 updated ISGPS classification [5], as a POPF requiring a change in postoperative management (grade B) or resulting in organ failure, reoperation, or death (grade C).

### 2.3. Surgical Technique

All procedures were performed by the same senior pancreatic surgeon as main operator, via an open approach according to the Traverso–Longmire (pylorus-preserving) or classic Whipple technique. Lymphadenectomy included anterior and posterior pancreaticoduodenal, pyloric region, hepatoduodenal ligament, common hepatic artery, and superior and inferior pancreatic head lymph nodes. Gastrointestinal reconstruction was performed according to the Child method, and a single-layer, duct-to-mucosa pancreatojejunostomy was performed in all cases. Details of the surgical technique have been previously described [14]. At the end of the procedure, two prophylactic intra-abdominal drains were placed around the pancreatic (left) and biliary (right) anastomoses [15].

### 2.4. Study Group

Serum lipase levels were measured preoperatively and on POD 1, 2, and 3. The laboratory normal range was 0–59 U/L (upper limit of normal [ULN]: 59 U/L). Patients were stratified into two groups based on serum lipase values: Elevated: serum lipase > 3× ULN (>177 U/L); Normal: serum lipase ≤ 3× ULN (≤177 U/L). The threshold of 3× ULN was chosen in accordance with the biochemical criterion used for the diagnosis of acute pancreatitis [7].

PCT was assessed in the central laboratory by an immunoluminometric assay. PCT plasma concentrations were measured using an automated Kryptor analyzer with time-resolved amplified cryptate emission (TRACE) technology (Kryptor PCT; Brahms AG, Hennigsdorf, Germany). The institutional normal PCT value was less than 0.5 ng/mL [12].

### 2.5. Statistical Analysis

Categorical variables were described as frequencies and percentages (%). Continuous variables were reported as median with range or as mean and standard deviation (SD), as appropriate. For univariate comparisons, the chi-square test or Fisher’s exact test was used for categorical variables. Continuous variables were analyzed using the *t*-test or Mann–Whitney U test, as appropriate. A *p*-value <0.05 was considered statistically significant.

A receiver operating characteristic (ROC) analysis was performed to assess the predictive performance of serum lipase for CR-POPF. Logistic regression analyses were applied to determine the occurrence of CR-POPF. Kendall’s Tau-b correlation coefficient was used to assess the association between PCT values on POD 3 and the severity of postoperative complications, POPF grade, and 90-day mortality. The combined predictive performance of elevated serum lipase and PCT above the identified cut-off was evaluated and reported in terms of sensitivity and specificity.

All statistical analyses were performed using SPSS version 26.0 for Mac (IBM Corp., Chicago, IL, USA).

## 3. Results

Of 196 patients who underwent PD during the study period, 115 had complete serum biomarker data across the first three PODs and were included in the analysis (median age = 68 years, BMI = 24.3 Kg/m^2^).

Pancreas head was mainly involved (*n* = 73, 63.5%), and the predominant histological diagnosis was adenocarcinoma (*n* = 87, 75.7%). All patients were evaluated by a multidisciplinary team, and most underwent upfront surgery (*n* = 106, 92.2%). Table 1 presents patients and tumors features.

### 3.1. Complications After Surgery

Major complications (defined as Clavien–Dindo ≥ IIIa) occurred in 37 patients (32.2%). Specifically, 22 (19.1%) experienced a CR-POPF: grade B in 12 patients (10.4%) and grade C in 10 patients (8.7%). Patients with CR-POPF had significantly higher rates of overall complications (100% vs. 55.9%, *p* < 0.001), major complications (90.9% vs. 18.3%, *p* < 0.001), longer hospital stays (26.5 ± 20.6 vs. 9.5 ± 3.4 days, *p* < 0.001), delayed right drain removal (9.1 ± 9.0 vs. 4.7 ± 1.8 days, *p* = 0.004) and delayed left drain removal (8.7 ± 11.5 vs. 4.6 ± 1.4 days, *p* = 0.025), higher hospital readmission rates (31.8% vs. 12.9%, *p* = 0.032), and increased 90-day mortality (31.8% vs. 7.5%, *p* = 0.002) compared to patients without CR-POPF (Supplementary Table S1).

### 3.2. Postoperative Serum Lipase Levels

Elevated serum lipase values were observed in 62 patients (53.9%) on POD 1, in 46 (40%) on POD 2, and in 26 (22.6%) on POD 3.

#### 3.2.1. Association with Postoperative Complications

Patients with elevated serum lipase were more likely to have soft pancreatic texture (*p* < 0.01) and small main pancreatic duct diameter (*p* < 0.01), and experienced higher rates of overall and major postoperative complications (Clavien–Dindo ≥ IIIa) across all three PODs (*p* < 0.05). Patients with persistently elevated serum lipase beyond POD 2 had longer ICU stays (*p* < 0.05) and prolonged hospitalization (*p* < 0.01). Elevated serum lipase on POD 3 was associated with increased 90-day mortality (*p* = 0.011) and a longer time to initiation of adjuvant therapy (*p* < 0.01) (Table 2).

#### 3.2.2. Association with Postoperative Fistula

**Figure 1:**
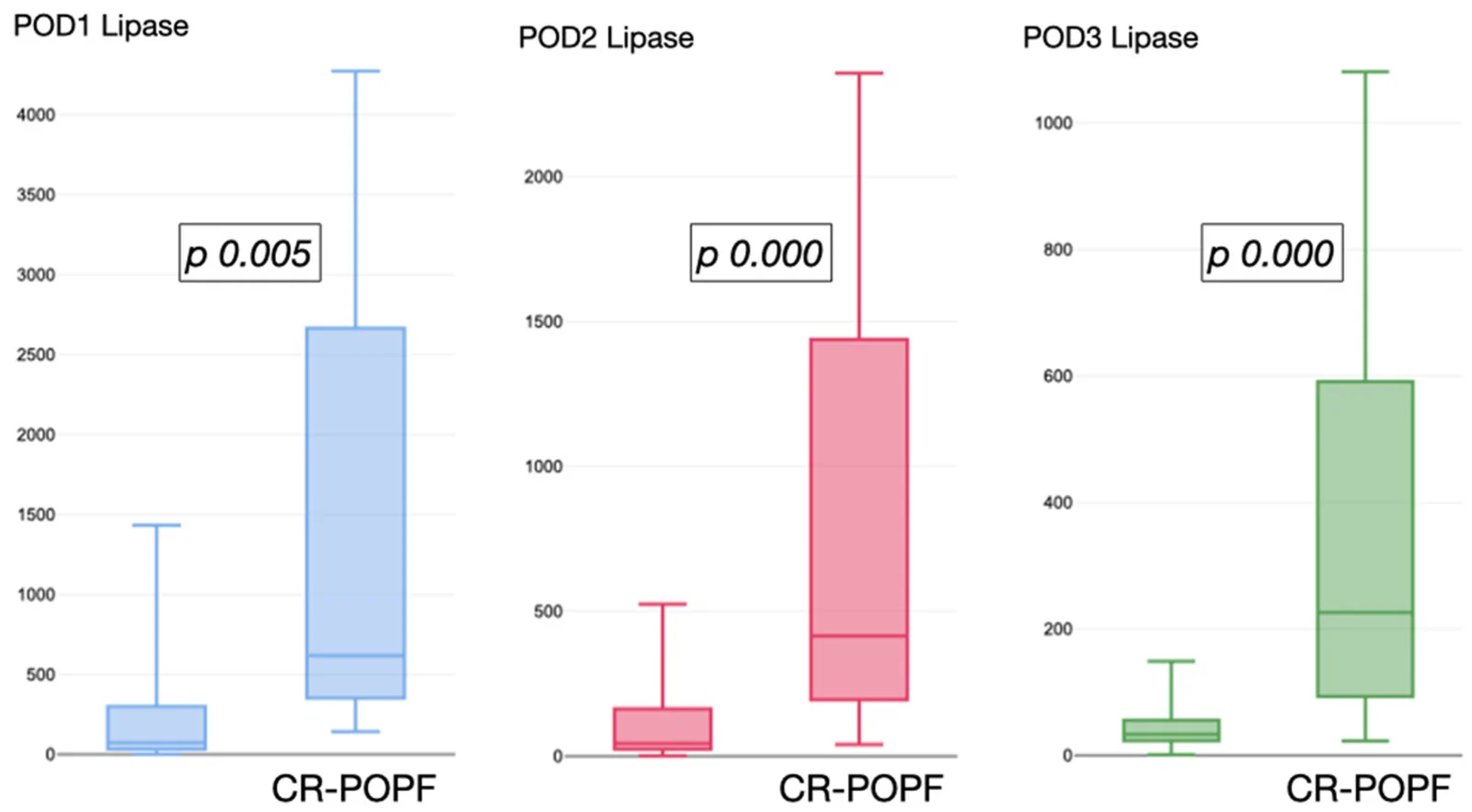
Distribution of serum lipase levels in first three postoperative days (PODs). Outliers were identified and excluded from the graphical representation (Tukey’s Fences).

Serum lipase levels were persistently higher in patients who developed CR-POPF compared to those who did not (*p* < 0.01), which, conversely, showed a progressive decrease in serum lipase levels, approximately halving from POD 1 to POD 2 and from POD 2 to POD 3 (Figure 1).

**Figure 2:**
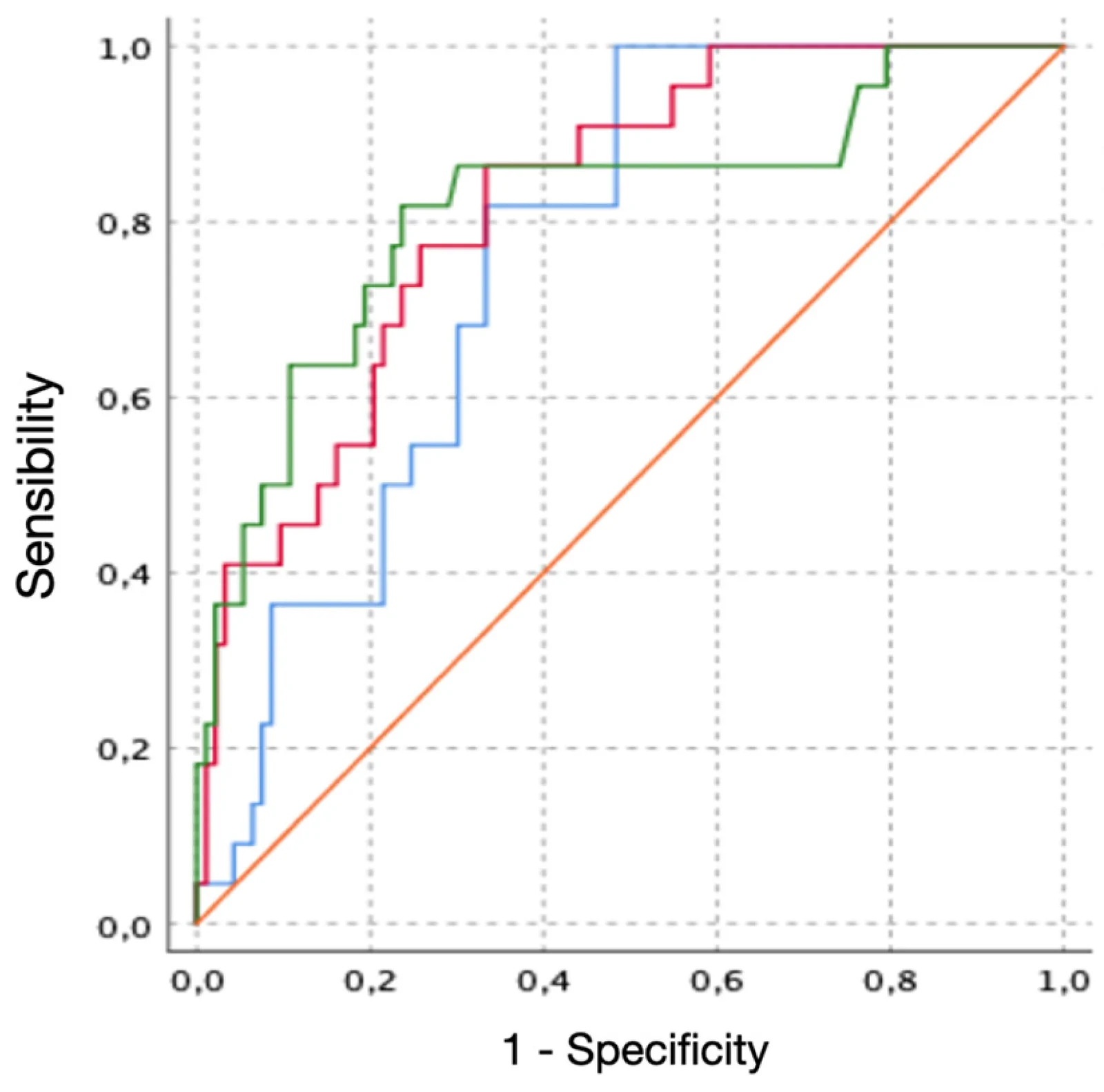
Accuracy of serum lipase in predicting CR-POPF. Blue line: lipase POD 1, red line: lipase POD 2, green line: lipase POD 3.

On ROC analysis, serum lipase values on POD 1, POD 2, and POD 3 achieved an AUC of 0.761 (95% CI 0.671–0.852, *p* = 0.001), 0.822 (95% CI 0.734–0.909, *p* = 0.001), and 0.817 (95% CI 0.707–0.928, *p* = 0.001), respectively (Figure 2). While sensitivity decreased from POD 1 to POD 3 (78% to 63.6%), specificity markedly increased (52.7% to 89.2%). The negative predictive value (NPV) remained high: 92.5% on POD 1, 92.7% on POD 2, and 93.3% on POD 3 (Supplementary Table S2).

On regression analysis, elevated serum lipase on POD 3 was the only independent predictor of CR-POPF (OR 6.89, 95% CI 1.607–29.534, *p* = 0.009), whereas elevated lipase on POD 1 (OR 1.71, 95% CI 0.347–8.435, *p* = 0.510) and POD 2 (OR 1.56, 95% CI 0.247–9.834, *p* = 0.637) did not reach statistical significance.

### 3.3. Preoperative Serum Lipase Levels

Preoperative concentrations were available in 77 patients (67%), with a mean value of 364 ± 587.4 U/L. The wide dispersion of values reflects the heterogeneity of underlying pancreatic and biliary pathology in the study population. No significant difference was observed between patients who developed CR-POPF (n = 9; 336.5 ± 549.9 U/L) and those who did not (n = 68; 533 ± 824.1 U/L; *p* = 0.640).

Similarly, the delta ratio between preoperative lipase and POD 3 showed no significant association with overall postoperative complications (*p* = 0.985), CR-POPF occurrence (*p* = 0.827), or 90-day mortality (*p* = 0.827).

### 3.4. Postoperative Procalcitonin

PCT levels on POD 3 were available for 94 patients (81.7%), with a mean value of 0.91 ± 2.9 ng/mL. PCT on POD 3 was significantly correlated with severe postoperative complications (Tau-b 0.245, *p* = 0.004), POPF grade (Tau-b 0.270, *p* = 0.001), and 90-day mortality (Tau-b 0.229, *p* = 0.007). The optimal PCT threshold for predicting CR-POPF, selected to maximize specificity, was 1.5 ng/mL (3× ULN; AUC 0.694), yielding a specificity of 90.8% (Table 3).

## 4. Discussion

Our study indicates that perioperative serum lipase might be a reliable early predictor of CR-POPF after PD. On POD 1, that marker is characterized by high sensitivity (78%), making it a useful screening tool to exclude the onset of severe complications. By POD 3, the test shifts toward high specificity (89.2%), effectively reducing false-positive rates and serving as a reliable confirmatory marker. In particular, persistently elevated serum lipase on POD 3 was independently associated with a nearly 7-times increased risk of CR-POPF (*p* = 0.009). Furthermore, the integration of serum lipase and PCT on POD 3 achieved higher specificity for early predicting the occurrence of CR-POPF, the appearance of which is primarily responsible for the delayed administration of adjuvant therapy and thus potentially influences oncological outcomes. Moreover, the clinical impact of CR-POPF extends beyond the immediate postoperative period: in a series of 123 patients undergoing pancreaticoduodenectomy for periampullary tumors, a worse grade of postoperative pancreatic fistula was significantly associated with delayed gastric emptying (*p* < 0.01), and both the presence of POPF and perineural infiltration emerged as independent prognostic factors for overall survival, the latter being in turn associated with DGE occurrence (*p* = 0.01) [16].

Conventionally, serum amylases have been extensively studied as an early marker of post-pancreatectomy complications (POPF and PPAP), while serum lipases have received comparatively less attention [11,14,17]. In a retrospective analysis of 212 patients, Chen et al. demonstrated that POD 1 serum lipase outperformed serum amylase in predicting CR-POPF, with an AUC of 0.801 versus 0.745 (*p* = 0.029) [18]. The authors also proposed a combined model of POD 1 serum lipase and POD 3 serum CRP, which showed good discrimination for CR-POPF (AUC 0.76) and an NPV of 94.3% in the low-risk group. More recently, Aghamaliyev et al. reported the largest cohort to date (471 patients undergoing PD and DP) comparing postoperative hyperlipasemia and hyperamylasemia [10]. In their multivariate analysis, only serum lipase the day of surgery remained an independent predictor of CR-POPF (OR 5.20, *p* < 0.001), further supporting the superiority of lipase over amylase in the early postoperative setting. Notably, the authors also described the kinetics of both enzymes, observing that while serum amylase returned to normal by POD 2, serum lipase remained elevated in the POPF group, suggesting a longer diagnostic window for lipase.

Our findings are consistent with this emerging evidence and extend it in two important directions. First, while Chen et al. focused on POD 1 and Aghamaliyev et al. on POD 0, we analyzed the trajectory of serum lipase across the first three PODs, demonstrating that the predictive accuracy increases over time, peaking at POD 2–3 (AUC 0.822 and 0.817, respectively) rather than at POD 0–1 (AUC 0.761). Second, the odd ratio (OR) for elevated lipase increased from POD 1 (1.71, not significant) to POD 3 (6.89, *p* = 0.009), which is higher than both Chen’s POD 1 OR of 4.78 and Aghamaliyev’s POD 0 OR of 5.20. This suggests that the later time point could more effectively discriminate patients with an ongoing pathological process rather than the transient enzyme release potentially associated with surgical manipulation [10,18]. Thus, a key finding of our study is the distinct temporal pattern of serum lipase in patients who developed CR-POPF compared to those who did not [12]. In the latter group, lipase levels progressively halved from POD 1 to POD 3, reflecting a physiological “washout” of the enzyme released during surgical manipulation of the pancreatic parenchyma. In contrast, patients with CR-POPF exhibited persistently elevated lipase levels, suggesting an ongoing inflammatory or ischemic process within the pancreatic remnant [4]. This observation is consistent with the pathophysiological model proposed by Connor, who hypothesized that a proportion of CR-POPF may result from postoperative pancreatitis rather than simple anastomotic leak [6]. The persistence of elevated serum lipase beyond POD 2 may serve as a biochemical surrogate of ongoing pancreatic inflammation, a concept that aligns with the ISGPS definition of the PPAP, which requires sustained hyperamylasemia for at least 48 h [7]. More broadly, pancreatic fistulas are not confined to the postoperative setting: ductal disruption in chronic pancreatitis may generate internal fistulas that track into the mediastinum and present as pleural effusion with minimal abdominal symptoms, occasionally bilaterally, and are therefore easily mistaken for primary thoracic disease [19]. Although clinically distinct from CR-POPF, these presentations share the same underlying mechanism, sustained leakage of pancreatic secretions from a disrupted duct, and underline how a biochemical signal of ongoing pancreatic damage may precede an overt clinical picture.

In a comprehensive review, Ismail and Bhayana highlighted several biochemical advantages of lipase over amylase [17]. First, lipase has a broader diagnostic window: serum lipase levels rise within 3–6 h of pancreatic injury, peak within 24 h, and remain elevated for up to two weeks, whereas amylase has a half-life of only 10–12 h with persistent elevation limited to 3–5 days. Second, lipase has greater organ specificity, being primarily produced by pancreatic acinar cells, while amylase can be elevated by multiple non-pancreatic sources including the salivary glands, intestinal tract, and various malignancies. Third, across multiple studies, the sensitivity of lipase for diagnosing acute pancreatitis ranges from 64% to 100%, compared to 45% to 87% for amylase, while specificity is comparable for both (92–99%). This longer diagnostic window of lipase likely explains why the AUC in our study was highest at POD 2–3 (0.822 and 0.817, respectively) rather than POD 1 (0.761): at the later time point, amylase has already normalized even in patients destined to develop CR-POPF, whereas lipase remains elevated, selectively identifying those with sustained parenchymal inflammation. Importantly, although we identify POD 3 as the pivotal cut-off, the use of POD3 serum lipase should not be interpreted as a recommendation to delay ERAS recommendations or drain removal strategies based on early postoperative assessment. The current trend toward early drain removal after PD relies on the assumption that low-risk patients can be identified within the first 48–72 h and may still benefit from early drain removal [20]. POD3 serum lipase should therefore be regarded as an additional risk-stratification: normal serum lipase on POD 3 (NPV 93.3%) could serve as a simple, inexpensive, and widely available tool to support this decision, complementing or even replacing drain fluid amylase in selected settings.

Notably, these results should also be framed within the context of established risk-stratification tools. The Fistula Risk Score (FRS) and its alternative version (a-FRS) provide a validated intraoperative estimate of CR-POPF risk based on gland texture, main pancreatic duct diameter, pathology and intraoperative blood loss, or body mass index in the a-FRS, with a discrimination ranging from 0.75 to 0.82 [21,22]. These tools are, however, static: they photograph the risk at the end of the operation and cannot capture what subsequently happens within the pancreatic remnant. Serum lipase, conversely, is a dynamic variable that reflects the biological evolution of the remnant over the first postoperative days. Consistently, Bannone et al. demonstrated that the sequential incorporation of early postoperative biological markers into the FRS progressively improved discrimination for CR-POPF, from an AUC of 0.82 for the FRS alone to 0.87 when postoperative hyperamylasemia was added and 0.90 when inflammatory C-reactive protein (CRP) was further included [23]. From a practical standpoint, we would therefore envisage a two-step strategy, in which the FRS defines the baseline anatomical and technical risk at the end of the operation, and POD 3 serum lipase, with or without PCT, re-stratifies the patient according to the actual behavior of the remnant [24].

Regarding PCT measure, its choice as second marker was not arbitrary. In a previous collaboration, postoperative PCT was shown to behave as a reliable exclusion marker for CR-POPF after PD, with a negative predictive value of approximately 90% from POD 2 onwards, and to increase the positive predictive value of drain fluid amylase from 71% to 81% when the two were combined [12]. The rationale for pairing PCT with serum lipase lies in the fact that the two markers interrogate different and sequential steps of the same pathological cascade: serum lipase is an organ-specific read-out of acinar injury and ductal leakage within the remnant, whereas PCT reflects the host systemic response to bacterial contamination of the resulting peripancreatic collection, which is precisely the event that converts a biochemical leak into a grade B or C fistula. In a meta-analysis of 20 studies, PCT and CRP showed comparable overall performance for POPF, with the most informative time points at POD 5 for PCT (AUC 0.87; sensitivity 0.84, specificity 0.74) and POD 4 for CRP (AUC 0.86; sensitivity 0.85, specificity 0.69) [25]. In single-center series, PCT outperformed CRP at earlier time points, with an AUC of 0.77 versus 0.53 on POD 1 and an AUC of 0.951 on POD 3 at a threshold of 2.10 ng/mL (sensitivity 88.2%, specificity 92.9%) [26,27]. Nevertheless, it should be acknowledged that PCT is not specific for a pancreatic source: in a prospective series, neither PCT nor CRP could discriminate patients with pancreatic fistula from those with postoperative infections of other origin [28]. This is fully consistent with our own findings, in which PCT alone performed only modestly as a standalone predictor (AUC 0.694, sensitivity 27.8%). Chen et al. proposed a dual-marker approach combining POD 1 serum lipase with POD 3 CRP, achieving an AUC of 0.76 and a PPV of 48.2% in the high-risk group [18]. Our model, by replacing CRP with PCT, yielded a PPV of 100%, achieving a markedly higher PPV. This difference may be explained by the higher specificity of PCT for bacterial infection and sepsis compared to CRP, which is a more general marker of systemic inflammation and can be elevated by multiple non-infectious postoperative stimuli [29].

Beyond pancreatic enzymes and PCT, other routinely available hematological parameters were previously investigated as early indicators of POPF. Platelets are of particular interest, being both acute-phase reactants and effectors of the inflammatory response, and their postoperative kinetics have therefore been proposed as a dynamic indicator of an evolving pancreatic complication. Papadoliopoulou et al. recently analyzed the ratio between postoperative and preoperative platelet counts from POD 1 to POD 9 in 133 patients undergoing pancreatic resection and found that a higher platelet count on POD 1 was associated with the occurrence of POPF (*p* = 0.04); notably, however, no association emerged with clinically relevant POPF [30]. Comparable inconsistencies characterize composite platelet-derived indices: whereas a low preoperative mean platelet volume-to-platelet count ratio has been reported as an independent predictor of CR-POPF (OR 13.91, *p* < 0.001) [31], the platelet-to-lymphocyte ratio showed no predictive value in two independent cohorts, with an AUC as low as 0.504 [32,33], and dynamic analyses of postoperative inflammatory markers identified the early rise in C-reactive protein, rather than any platelet-derived index, as the most informative signal [34]. These observations reinforce the rationale of our approach. Platelet kinetics reflect a generic systemic acute-phase response and are therefore exposed to several non-pancreatic confounders, transfusion, sepsis of any origin, surgical manipulation and haemodilution, whereas serum lipase provides a direct, organ-specific read-out of acinar injury within the remnant. This may explain why platelet-based signals appear to track POPF as a whole but lose discriminative power precisely where it matters clinically, namely in identifying grade B/C fistulas. POD 3 serum lipase and platelet dynamics should therefore be regarded as conceptually different and potentially complementary layers of information, whose combination deserves dedicated evaluation.

Ultimately, the 90-day mortality rate observed in our cohort is consistent with more recent single-center data from Luu et al., who reported a 39.1% rate in patients who experienced a grade C POPF after 722 PD [35]. In addition, the high prevalence of soft pancreatic texture (63.5%) and small pancreatic duct diameter (39.1%) identifies our cohort as a population at inherently high fistula risk. These data are in line with recent international evidence from the PancreasGroup.org study, which reported a failure-to-rescue rate of 21.9% after PD, confirming that severe complications, once established, carry a substantial mortality risk even in experienced centers [3].

The retrospective, single-center design limits the generalizability of the findings, and the exclusion of 81 patients due to incomplete data may have introduced a selection bias. We acknowledge a partial overlap between the present cohort and the series previously reported by our group [12]. That analysis addressed a different question: it was confined to PCT and drain fluid amylase, did not consider serum lipase at any time point, and was designed to identify a rule-out threshold (PCT 0.5 ng/mL) for excluding CR-POPF as a primary endpoint. The two analyses therefore provide complementary rather than redundant information. On the other hand, patients with incomplete data could potentially differ from those included in the analysis, limiting the generalizability of our findings. More specifically, given the incomplete availability of PCT measurements, the observed diagnostic performance requires external validation in larger independent cohorts. Moreover, although serum lipase showed a high NPV in our cohort, this parameter is strongly influenced by the prevalence of CR-POPF and may therefore vary across different patient populations and clinical settings. Unlike Chen et al., we did not directly compare serum lipase with serum amylase within the same cohort, which limits the conclusions regarding the superiority of one marker over the other. As previously stated, the FRS was not formally computed in the present cohort: intraoperative blood loss was below 400 mL in the vast majority of patients and 75.7% had a ductal adenocarcinoma, so that two of the four FRS components would have contributed minimally and the score would have been almost entirely driven by gland texture and duct diameter, both already reported and both strongly associated with postoperative hyperlipasemia (*p* < 0.01, Table 2). A formal head-to-head comparison, and the derivation of an integrated FRS-plus-lipase model, will require larger, prospective, and preferably, multi-center cohorts [35]. Moreover, the threshold of 3× ULN, while consistent with the Atlanta classification for acute pancreatitis [36], has not been independently validated for the prediction of CR-POPF and may require optimization in future studies. Ultimately, we advocate to integrate our findings with a more detailed molecular characterization of tumors [37].

## 5. Conclusions

In conclusion, our results suggest that POD 3 serum lipase, with or without PCT, may contribute to postoperative risk stratification after PD. In patients with a normal POD 3 serum lipase, the high negative predictive value makes CR-POPF unlikely, an observation that could be integrated into enhanced recovery pathways, although this hypothesis requires prospective validation. Conversely, patients with persistently elevated POD 3 serum lipase carry a substantially increased risk of CR-POPF and may warrant closer clinical and biochemical surveillance. Since no management strategy, including drain removal, cross-sectional imaging or antimicrobial treatment, was prospectively tested in the present study, our findings should be regarded as hypothesis-generating rather than as a management recommendation.

## Figures and Tables

**Table 1:** Patients and tumor characteristics.

	Overall (*n* = 115)
Age in years median (range)	68 (38–85)
Gender, *n* (%) Male Female	63 (54.8) 52 (45.2)
BMI, kg/m^2^ median (range)	24.3 (17.6–38.5)
ASA score median (range)	2 (1–3)
Comorbidities, *n* (%) Cardiovascular disease * Diabetes Severe kidney failure Respiratory disease Smoking Previous neoplasm	48 (41.7) 15 (13) 1 (9) 11 (9.5) 21 (18.3) 11 (9.5)
Tumor site, *n* (%) Pancreas Vater’s Ampulla Duodenum Common bile duct	73 (63.5) 23 (20) 3 (2.6) 16 (13.9)
Histology, *n* (%) Ductal adenocarcinoma Cholangiocarcinoma Neuroendocrine tumor IPMN Others	87 (75.7) 13 (11.3) 6 (5.2) 3 (2.6) 6 (5.2)
Neoadjuvant, *n* (%)	9 (7.8)
Surgical technique, *n* (%) Whipple *n* (%) Pylorus-preserving *n* (%)	38 (33) 77 (67)
Operative time, minutes median (range)	360 (135–600)
Duct size, mm median (range)	4.2 (1–10)
Soft pancreatic texture, *n* (%)	73 (63.5)

* Including hypertension under treatment. Acronym, BMI: Body mass index, ASA: American Society of Anesthesiologists classification grade, IPMN: Intraductal Papillary Mucinous Neoplasm (IPMN).

**Table 2:** Features in patients with normal or elevated serum lipase concentrations.

	POD 1 Lipase			POD 2 Lipase			POD 3 Lipase		
	Normal (*n* = 53)	Elevated (*n* = 62)	*p*	Normal (*n* = 69)	Elevated (*n* = 46)	*p*	Normal (*n* = 89)	Elevated (*n* = 26)	*p*
Main duct size < 3 mm, *n* (%)	41 (77.4%)	29 (41.4%)	**0.001**	52 (75.4%)	18 (39.1%)	**0.001**	64 (71.9%)	6 (23.1%)	**0.001**
Soft pancreatic texture, *n* (%)	26 (49.1%)	47 (75.8%)	**0.003**	35 (50.7%)	38 (82.6%)	**0.001**	48 (53.9%)	25 (96.2%)	**0.001**
EBL mL (mean ± SD)	345 ± 184	306 ± 146	0.247	324 ± 169	324 ± 162	0.989	321 ± 173	336 ± 137	0.658
Complications, *n* (%)	25 (47.2%)	49 (79%)	**0.001**	38 (55.1%)	36 (78.3%)	**0.011**	51 (57.3%)	23 (88.5%)	**0.004**
Severe complications, *n* (%)	12 (22.6%)	25 (40.3%)	**0.043**	13 (18.8%)	24 (52.2%)	**0.001**	18 (20.2%)	19 (73.1%)	**0.001**
CR-POPF *n* (%)	4 (7.5%)	18 (29%)	**0.004**	5 (7.2%)	17 (37%)	**0.001**	8 (9%)	14 (53.8%)	**0.001**
Length of hospital stay in days (mean ± SD)	16.9 ± 14.5	23.3 ± 20.9	**0.057**	16.1 ± 12.3	27 ± 23.8	**0.003**	16.3 ± 12	34.8 ± 28.2	**0.001**
ICU stay (mean ± SD)	1.24 ± 1	1.91 ± 4.6	0.298	1.2 ± 0.9	2.1 ± 5.2	0.236	1.2 ± 0.8	2.8 ± 6.7	**0.039**
Right drainage removal (mean ± SD)	6.4 ± 5.7	8.4 ± 8.8	0.174	6.6 ± 5.3	8.8 ± 10.1	0.229	6.5 ± 5.5	11.1 ± 12.5	**0.015**
Left drainage removal (mean ± SD)	7.1 ± 12.6	6.9 ± 3.7	0.914	5.7 ± 5.3	9 ± 12.7	0.122	5.6 ± 4.7	12.3 ± 17	**0.002**
Percutaneous or endoscopic drainage, *n* (%)	9 (17%)	19 (30.6%)	0.089	11 (15.9%)	17 (37%)	**0.010**	16 (18%)	12 (46.2%)	**0.003**
Angiographic procedure for bleeding, *n* (%)	1 (1.9%)	6 (9.7%)	0.082	3 (4.3%)	4 (8.7%)	0.339	4 (4.5%)	3 (11.5%)	0.186
Reoperation, *n* (%)	3 (5.9%)	8 (12.9%)	0.210	4 (6%)	7 (15.2%)	0.103	4 (4.6%)	7 (26.9%)	**0.001**
90-day mortality, *n* (%)	6 (12%)	8 (12.9%)	0.886	7 (10.6%)	7 (15.2%)	0.468	7 (8.1%)	7 (26.9%)	**0.011**
90-day hospital readmission *n* (%)	3 (5.7%)	16 (25.8%)	**0.004**	7 (10.1%)	12 (26.1%)	**0.024**	12 (13.5%)	7 (26.9%)	**0.105**

Acronym, POD: Postoperative day; EBL: Estimated blood loss; ICU: Intensive Care Unit.

**Table 3:** Combination of PCT and serum lipase in predicting the CR-POPF.

Title 1	Value	Sensitivity	Specificity	PPV	NPV	PLR	NLR
PCT	>1.5 ng/mL (3 ULN) in POD 3	27.8%	90.8%	41.7%	84.2%	3.02	0.80
Combined lipase and PCT	lipase > 3 ULN and PCT > 3 ULN in POD 3	37.5%	100%	100%	92.1%	-	0.62

Acronym, PPV: Positive predictive value; NPV: Negative predictive value; PLR: Positive Likelihood Ratio; NLR: Negative Likelihood Ratio; PCT: Procalcitonin; ULN: Upper Limit Normal.

## Data Availability

The data presented in this study are available on request from the corresponding author due to Data Protection Policy.

## Supplementary Materials

The following supporting information can be downloaded at https://www.mdpi.com/article/10.3390/cancers18172821/s1, Supplementary Table S1: Postoperative complications; Supplementary Table S2: Accuracy of serum lipase in predicting CR-POPF.

## Abbreviations

The following abbreviations are used in this manuscript:

PD	Pancreaticoduodenectomy
POPF	Postoperative pancreatic fistula
CR-POPF	Clinically relevant POPF
ISGPS	International Study Group of Pancreatic Surgeons
POAP	Postoperative acute pancreatitis
PPAP	Post-pancreatectomy acute pancreatitis
POD	Postoperative day
PCT	Procalcitonin
ULN	Upper limit of normal
CRP	C-reactive protein
