# Supplementary material for: Optimal Timing of Serum Lipase for Early Prediction of Clinically Relevant Pancreatic Fistula After Pancreaticoduodenectomy
Source: Cancers (Basel). 2026 Sep 1;18(17):2821. doi: 10.3390/cancers18172821 (PMC13564947; doi:10.3390/cancers18172821)
Supplement: Supplementary file 1 [file cancers-18-02821-s001.zip › cancers-4524382-supplementary.pdf]

Supplementary Table S1. Postoperative complications.

	Overall (n = 115)	Absence of CR-POPF (n = 93)	CR-POPF (n = 22)	p
Complication, n (%)	74 (64.3%)	52 (55.9%)	* 22 (100%)	**0.001**
Severe complication (Clavien-Dindo ≥IIIa), n (%)	37 (32.2%)	17 (18.3%)	* 20 (90.9%)	**0.001**
Length of hospital stay (mean±SD)	20.36±18.44	9.49±3.42	26.5±20.55	**0.001**
ICU stay (mean±SD)	1.60±3.45	1.11±03.45	1.87±4.26	0.284
Right drainage removal (mean±SD)	7.38±7.45	4.69±1.78	9.06±9.02	**0.004**
Left drainage removal (mean±SD)	7.04±9.13	4.55±1.43	8.70 + 11.45	**0.025**
Percutaneous or endoscopic drainage, n (%)	28 (24.3%)	12 (13.1%)	16 (72.7%)	**0.001**
Angiographic procedure for bleeding, n (%)	7 (6.1%)	2 (2.1%)	5 (22.7%)	**0.001**
Reoperation, n (%)	11 (9.7%)	7 (7.5%)	4 (18.2%)	0.122
Mortality up to 90 days after surgery, n (%)	14 (12.2%)	7 (7.5%)	7 (31.8%)	**0.002**
90-day Hospital readmission, n (%)	19 (16.5%)	12 (12.9%)	7 (31.8%)	**0.032**
Start of adjuvant therapy within 10 weeks n (%)	60 (52.2%)	54 (58.1%)	6 (27.3%)	0.070

* Excluding CR-POPF. Acronym, ICU: Intensive Care Unit.

Supplementary Table S2. Accuracy of serum lipase in predicting CR-POPF.

Variable	Value	Sensitivity	Specificity	PPV	NPV	AUC	p	95% Cl Lower	95% Cl Upper
POD 1 lipases	> 177 U/l (3 ULN)	78%	52.7%	29%	92.5%	0.761	**0.001**	0.671	0.852
POD 2 lipases	> 177 U/l (3 ULN)	77.3%	68.8%	36.6%	92.7%	0.822	**0.001**	0.734	0.909
POD 3 lipases	> 177 U/l (3 ULN)	63.6%	89.2%	53.4%	93.3%	0.817	**0.001**	0.707	0.928

Acronym, POD: post-operative day; PPV: Positive Predictive Value; NPV Negative Predictive Value; AUC: Area Under Curve.
